# New Insights into Drug Development via the Nose-to-Brain Pathway: Exemplification Through Dodecyl Creatine Ester for Neuronal Disorders

**DOI:** 10.3390/pharmaceutics18010080

**Published:** 2026-01-07

**Authors:** Henri Benech, Victoria Flament, Clara Lhotellier, Camille Roucairol, Thomas Joudinaud

**Affiliations:** Ceres Brain Therapeutics, Institut du Cerveau ICM, Hôpital de la Pitié-Salpêtrière, 47 Bd de l’Hôpital, 75013 Paris, France

**Keywords:** intranasal, Nose-to-Brain, blood–brain barrier, drug delivery, neurological diseases, dodecyl creatine ester, creatine transporter deficiency, amyotrophic lateral sclerosis

## Abstract

Brain disorders remain a major global health challenge, highlighting the urgent need for innovative therapeutic strategies and efficient drug-delivery approaches. Among alternative routes, intranasal administration has garnered significant interest over recent decades, not only for its systemic delivery but also for its unique ability to bypass the bloodstream and the blood–brain barrier via the Nose-to-Brain (NtB) pathway. While numerous reviews have explored the opportunities and challenges of this route, industrial considerations—critical for successful clinical implementation and commercial development—remain insufficiently addressed. This review provides a comprehensive and critical assessment of the NtB pathway from a drug development and chemistry, manufacturing, and controls perspective, addressing key constraints in pre-clinical–clinical extrapolation, formulation design, device selection, dose feasibility, chronic safety, and regulatory requirements. We also discuss recent advances in neuronal targeting mechanisms, also with a focus on the role of trigeminal nerves. Dodecyl creatine ester (DCE), a highly unstable in plasma creatine prodrug developed by Ceres Brain Therapeutics, is presented as an illustrative case study. Delivered as a nasal spray, DCE enables direct neuronal delivery, exemplifying the potential of the NtB pathway for disorders characterized by neuronal energy deficiency, including creatine transporter deficiency and mitochondrial dysfunction. Overall, the NtB pathway—or, more precisely, the “Nose-to-Neurons” pathway—offers distinct advantages for unstable molecules and metabolic supplementation, particularly in neuron-centric diseases. Its successful implementation will depend on rational molecule design, optimized nasal formulations, appropriate devices, and early integration of industrial constraints to ensure feasibility, scalability, and safety for long-term treatment.

## 1. Introduction

Brain disorders affect millions of people worldwide, highlighting the critical need for effective treatments. To enhance current treatments or develop new ones, it is essential for researchers and drug developers to push forward the developments of brain-targeting drugs, which will ultimately benefit patients. Alongside the development of novel drugs, addressing drug delivery is crucial and can play a key role in the success of future clinical trials. Interest in the nasal route for drug delivery began to rise in the 1980s, and two comprehensive reviews by Scheen et al. in 1988 [1] and Wüthrich et al. in 1989 [2] highlighted the potential of this administration route. Since then, the development of nasal drug products has gained significant interest, initially as a means to deliver drugs into the bloodstream. This interest was built on foundational studies from the early 2000s showing that intranasally administered agents can reach the brain via olfactory and trigeminal pathways [3,4,5,6] These seminal studies established the mechanistic principles that underpin modern translational and formulation-driven developments. More recently, nasal drug development has aimed to directly target the brain by bypassing both the bloodstream and the blood–brain barrier (BBB) via what has been termed the Nose-to-Brain (NtB) pathway. Several reviews have been published over the last 10–15 years on the advantages and challenges of this pathway [7,8,9,10]. While the NtB route presents promising opportunities, its implementation faces challenges such as dose limitations extrapolation from animals to humans, and inter-individual variability. Few reviews discuss the value of the NtB pathway from an industrial perspective [7,10,11], which is of major importance for drug developers since most published manufacturing processes are difficult to industrialize and may even be impossible to implement on a commercial scale. Therefore, it is crucial to consider the limitations related to industrial development for future medications using the NtB administration route.

The development strategy for drugs targeting the brain through the NtB pathway includes exploratory preclinical pharmacology, toxicology, and efficacy studies in animals, followed by similar studies in humans, alongside Chemistry, Manufacturing, and Controls (CMC) for both early development and commercialization. Since drug distribution from the site of deposition to the brain cannot be directly monitored in clinical settings, and plasma concentrations poorly reflect brain exposure of a drug that follows the NtB pathway, pharmacokinetic–pharmacodynamic relationships are often lacking, complicating clinical development.

This review aims to synthesize and critically evaluate the NtB pathway as a strategy for brain drug delivery, highlighting its advantages, and the challenges that remain from an industrial perspective. In addition, we provide new insights into brain cell targeting and the role of trigeminal nerves. These concepts are illustrated through example of Dodecyl creatine ester (DCE), a creatine prodrug [12] currently in clinical development.

Developed by Ceres Brain Therapeutics, DCE is administered as a nasal spray, enabling the direct delivery of creatine to neurons. This approach holds promise for neurological disorders, including creatine transporter deficiency syndrome, and mitochondrial diseases linked to neuronal energy deficiency. Due to its high instability in the bloodstream, DCE serves as a compelling model to demonstrate the efficacy and specificity of the NtB delivery route [13].

## 2. Considerations of Brain Targeting for Neurotherapeutics

After entering the bloodstream, drugs are distributed to different organs based on their physicochemical properties, such as logP or pKa, resulting in concentration ratios between organs and blood. For a systemic brain-targeting drug (e.g., so-called neurodrugs), the objective is to achieve a high ratio between brain and blood concentrations, ensuring that the drug reaches the brain in sufficient quantities to exert its effect and at a rate that is compatible with the indication, e.g., analgesics require rapid dissemination in the brain [14]. The most frequently used routes for drug administration are oral or intravenous (IV), resulting in high systemic exposure but risking nonspecific biodistribution and subsequent toxicities. Achieving effective drug concentrations in the target organ with a therapeutically acceptable dose while minimizing off-target effects due to nonspecific tissue distribution remains a significant challenge in the field of neurodrugs [15].

After administration through the conventional oral route, a neurodrug has to overcome several obstacles before reaching its target. These obstacles include metabolism by intestinal enterocyte cells (via enzymes such as cytochrome P450), efflux proteins (e.g., P-gP), and enzymes in the bloodstream (e.g., esterases). These are followed liver metabolism—with its first-pass effect and enzymes such as cytochrome P450 and glucuronyl-transferases—and ultimately, the BBB. The barrier posed by enterocytes is only present for oral administration: using intravenous, sublingual, rectal, subcutaneous, or intramuscular administration help in bypassing the first-pass effect. However, regardless of the route of administration, once the neurodrug enters the bloodstream, it must overcome both blood enzymes and the BBB to reach the brain.

The BBB presents a formidable challenge, underscoring the importance of developing innovative drug delivery systems to bypass or penetrate it. It is a highly selective semipermeable biological membrane that separates the circulating blood from the brain’s extracellular fluid. Composed of specialized endothelial cells, astrocytes, and pericytes, the BBB and particularly the junctions between cells tightly regulates the passage of drugs into the brain, maintaining brain homeostasis and protecting it from potentially harmful substances. In addition to its structural components, the BBB features influx and efflux transporters that regulate the movement of molecules into and out of the brain. Influx transporters facilitate the uptake of essential nutrients, and neurotransmitters. These include glucose transporter proteins (GLUTs), amino acid transporters, and monocarboxylate transporters (MCTs), which facilitate the entry of essential nutrients and molecules into the brain [16]. Conversely, efflux transporters actively pump potentially harmful substances and excess neurotransmitters out of the brain, maintaining brain homeostasis and protecting against neurotoxicity. These include P-glycoprotein (P-gp) [17], breast cancer resistance protein (BCRP) [18], and multidrug resistance-associated proteins (MRPs) [17]. These transporters play a crucial role in drug delivery to the brain and contribute to the selective permeability of the BBB but limit the efficacy of drugs intended to target the central nervous system. Dysfunction of the BBB is implicated in various neurological disorders, highlighting its critical role in brain health and function and such dysfunction may also modify its permeability to drugs [19]. Thus, crossing the BBB, whether in individuals with an intact or dysfunctional BBB, represents a significant challenge for neurodrugs.

These constraints have challenged drug developers in the past and continue to do so, and approaches to enhance drug transport across the BBB include: (1) synthesis of small molecules with efficacious activity on the target, (2) chemical modification of drug molecules to enhance lipophilicity, thereby increasing apolarity and enabling passive permeation, bypassing efflux transporters [20], (3) leveraging endogenous transport systems, such as receptor-mediated transcytosis, to also bypass the efflux transporters [20], (4) nanoparticle-based drug delivery systems, exploiting size-dependent permeability or surface modifications to facilitate BBB penetration [21]; (5) ultrasound-mediated techniques, including focused ultrasound and microbubble-assisted sonoporation, which temporarily disrupts the BBB to allow transient drug passage [22]; (6) emerging technologies such as cell-penetrating peptides and exosome-based delivery systems [23,24]; (6) alternative routes of administration that avoid the BBB, such as intrathecal, intracerebroventricular delivery, and the NtB pathway via intranasal dosing.

The intrathecal route is used for a limited number of drugs, e.g., BRINEURA^®^, an enzyme replacement therapy used to treat CLN2 Batten disease by compensating for the deficiency of the tripeptidyl peptidase 1 enzyme in the brain. The clinical value of this route lies in the delivery of drugs directly into the cerebrospinal fluid [3], but concerns about safety and the considerable burden it places on caregivers have limited its widespread use for many neurodrugs.

**Figure 1:**
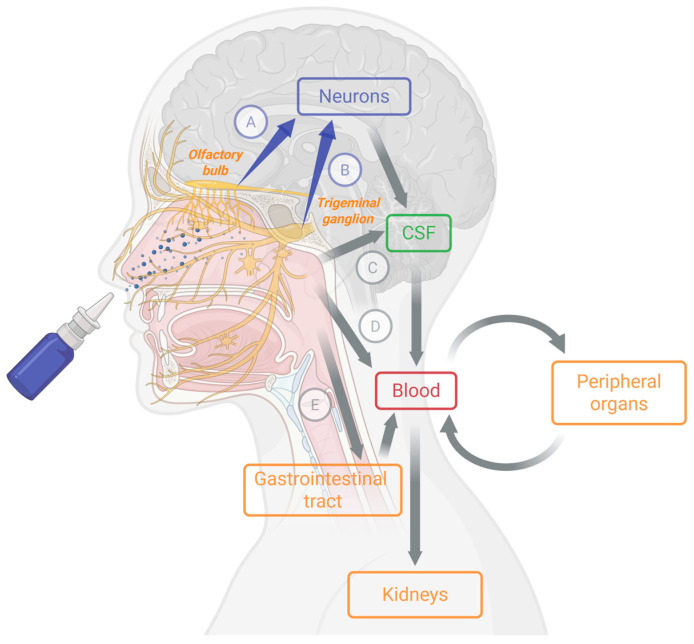
Different routes of drug transport after intranasal administration: (A) Gradual diffusion along the anatomical projections of the olfactory bulb (*Bulbus olfactorius*). (B) Gradual diffusion along the anatomical projections of the trigeminal ganglion (*Ganglion trigeminale*). (C) Entry into the CSF via olfactory and trigeminal nerve pathways. (D) Absorption into the bloodstream through the nasal microvasculature. (E) Absorption into the bloodstream via the gastrointestinal tract after swallowing.

While these strategies show promise in overcoming the BBB’s defenses, ongoing research is crucial to evaluate their efficiency, safety, and clinical applicability for the delivery of neurodrugs. Intranasal administration has demonstrated significant efficacy, largely attributed to neural transport mechanisms, whereby drugs migrate along the nerve pathways to access specific regions within the central nervous system. The NtB pathway constitutes one of the potential routes for a drug after its administration in the nose, which include: (i) absorption into the bloodstream through the numerous microvessels in the nasal cavity, (ii) swallowing, after reaching the throat through the back of the nasal cavity, (iii) the NtB pathway via the olfactory and trigeminal nerves and subsequent passage into the cerebrospinal fluid (CSF). Inhalation into the lungs is very unlikely if the drug is sprayed in the form of particles larger than 10 µm. This pathway is illustrated in Figure 1.

The non-invasive nature of this route of administration is a key benefit, reducing patient discomfort and enhancing treatment compliance, particularly in populations where invasive procedures are less desirable, such as pediatric or geriatric patients.

The growing interest in the intranasal route of administration for neurodrugs is reflected in the recent scientific literature. For research with a keyword of ‘Nose-to-brain’, 9 articles were published in 2010, 35 in 2015, 106 in 2020, and 164 in 2025, demonstrating a significant increase in publications on this topic in recent years. The literature offers a wealth of strategies for the development of drugs intended for nasal administration. Information on optimal physicochemical properties, the most effective formulation techniques, and the most appropriate devices has been extensively documented for over 30 years [25]. However, given the complexity of the NtB pathway and the need for dedicated, specialized development efforts, regularly reviewing the existing literature is insufficient, and proposing new strategies is essential.

## 3. Opportunities and Challenges of the Intranasal Delivery Route for Improving the Performance of Neurodrugs

### 3.1. Physiological Aspects of the NtB Pathway

The nasal cavity is a dynamic environment where variation in airflow, pH, mucus composition, and nasal mucociliary clearance influence drug deposition, retention, and absorption. Nasal mucociliary clearance moves the mucus layer toward the nasopharynx at a rate of ~5 mm/min, with a transit time of 15–20 min [26]. Nasal mucus is composed of water, salts, proteins and glycoproteins that trap particles, pathogens, and allergens. Its slightly acidic pH, which has been reported to be between 5.0–7.0, and varies with region, air temperature, sleep, emotions, and food intake [2,27].

The human nasal cavity (around 160 cm^2^) includes the olfactory epithelium (around 10 cm^2^ [5%]) [7,8,28] and the respiratory epithelium, which lines most of the nasal cavity and is highly vascularized. The anatomy of the nasal cavity, including its nerves and blood vessels, has extensively presented elsewhere [29].

The NtB pathway mainly involves the olfactory and trigeminal nerves. Olfactory sensory neurons project from the nasal epithelium through the cribriform plate to the olfactory bulb, creating a direct neuronal connection between the nasal cavity and the central nervous system. In parallel, trigeminal nerve fibers innervate both the respiratory and olfactory mucosa and project centrally to the trigeminal ganglion and brainstem nuclei. Following intranasal administration, compounds may reach the central nervous system (CNS) via both intracellular neuronal transport and extracellular pathways, including perineural and perivascular diffusion along these nerves, thereby enabling access to central brain structures, as demonstrated in preclinical studies [13]. These pathways have been extensively presented elsewhere [6,30].

The olfactory region was first described as the principal entry route to the brain of the olfactory nerves [31] although the less extensively studied trigeminal nerves also play a significant role in drug delivery to the CNS [32]. After intranasal administration, drugs contact the olfactory and trigeminal nerve terminals in the mucus and can access the subarachnoid space surrounding the pons or the region near the olfactory bulb. Intranasal dopamine crosses the nasal pseudoepithelium along trigeminal nerve branches [33] and trigeminal transport of the glucagon derivative GLP-2 to the dorsomedial nucleus of the thalamus and the hippocampus has been shown in mice [34]. Once in the CNS, drug distribution involves synaptic transfer between neurons, extracellular diffusion, and bulk flow of CSF [35].

Paracellular diffusion enables extracellular delivery from both the olfactory and respiratory epithelia, with the trigeminal nerves specifically facilitating transport for the latter. In the case of olfactory nerve fibers, extracellular transport can take place within a direct, continuous channel from the olfactory epithelium to the olfactory bulb. This transport may be further supported by the rapid regeneration of olfactory sensory neurons [36].

Radiolabeled IGF-I studies have confirmed dual olfactory/trigeminal delivery to the CNS, including the olfactory bulb and brainstem [6,37] and manganese-enhanced magnetic resonance imaging has further demonstrated neuronal uptake [31].

Emerging evidence suggests that brain cellular uptake predominantly favors neurons [13] and is initiated by the axonal transport and synaptic exocytosis to other brain regions through neural projections [30]. For example, insulin with L-penetrating peptide accumulates in hippocampal neuronal cells [38] promoting survival and protection of neurons [38,39]. Similarly, intranasal DCE has been shown to deliver creatine to cortical neurons in macaques [13] and a glucagon derivative has shown antidepressant-like effects comparable to intracerebroventricular administration [34,40].

### 3.2. Critical Aspects of NtB Delivery

NtB delivery depends on multiple interacting factors, including the physicochemical properties of the drug, the relative contributions of intracellular neuronal and extracellular perineural transport mechanisms, and formulation design—particularly excipient selection (including preservatives)—which critically influences residence time, local tolerability, and stability. Additional determinants include nasal metabolism, inter-individual anatomical variability, and mucociliary clearance. Collectively, these factors shape both efficacy and safety and must be integrated early into formulation and development strategies.

More precisely, small, lipophilic, non-ionized drugs are generally expected to be more easily absorbed from the nasal cavity [41] but this appears only to apply to systemic absorption. An extensive literature review has shown that, for a wide range of compounds, the efficiency of the NtB pathway was surprisingly unrelated to either the molecular weight or the LogD at pH 7.4 [42]. This could be explained by several factors. First, transport through the olfactory and trigeminal nerve endings differs from classical transmembrane absorption, which occurs via basal nasal cells before reaching the systemic circulation.

The type of transport mechanisms influences delivery: axonal transport favors small lipophilic drugs, while extracellular pathways accommodate hydrophilic molecules across a wide range of molecular weights [43]. As a result, small, lipophilic molecules can reach the CSF and olfactory bulb rapidly, peaking around 15 min after nasal administration, whereas hydrophilic compounds show slower onset [7]. Finally, olfactory and trigeminal pathways collectively transport molecules with diverse physicochemical properties [37] and caution is needed when interpreting the relationship between a drug’s physicochemical characteristics and its NtB efficiency.

Extensive evidence highlights drug formulation to be a critical determinant of NtB delivery. Particle-based formulations are generally more effective than gels or solutions for transporting drugs via the NtB pathway [42] (less information is available for powder formulations). Microemulsions and nanoemulsions are particularly promising due to their small globule size and lipophilic properties. These emulsions are isotropic, transparent, low-viscosity colloidal systems stabilized by surfactants [44] and contain inactive ingredients such as oil (e.g., isopropyl myristate, triglycerides, soybean oil) and water. A wide range of surfactants/co-surfactants is available for both commercial and scientific research use, but only oleyl polyethylene glycol (PEG), PEG 8 stearate, PEG 3350, PEG 400, PEG 6000, polysorbate 20, polysorbate 80, propylene glycol and sorbitan trioleate are allowed for intranasal use [45]. It should be noted, however, that common co-solvents such as glycols, small amounts of alcohol, transcutol, medium chain glycerides, and labrosol can irritate the nasal mucosa, and even low levels of preservatives or taste-masking agents derived from essential oils can contribute to local irritation [25]. Surfactants, in particular, may interact with biological membranes and induce dose-dependent tissue damage [46]. Nevertheless, these components are sometimes necessary, particularly flavoring agents that can mask an unpleasant taste or odor [47]. Some permeation enhancers, however, such as bile salts, fatty acids, cyclodextrins or chitosan mainly facilitate absorption into the bloodstream and, to our knowledge, there is no clear evidence that they truly enhance NtB transport: given their potential local safety issues, their use in long-term treatments should be approached with caution.

Both residence time and local tolerance are key determinants of the efficiency of intranasal delivery. Humectants, mucoadhesive polymers, cosurfactants, and viscosity enhancers can prolong residence time, though tolerance must be carefully monitored. Mucoadhesive gels may further improve absorption and transfer [48]. Regulatory status, particularly whether these excipients are classified as Generally Recognized As Safe (GRAS) by regulatory authorities, should also be carefully considered. However, viscosity is a key factor in nasal drug delivery—moderate viscosity prolongs contact between drug and nasal mucosa, improving potential absorption. Agents such as xanthan gum and methylcellulose serve as viscosity enhancers for liquid formulations, while mucoadhesive systems, liposomes, microspheres, and nanoparticles can further extend residence time and promote both transport and systemic absorption [49]. However, excessive viscosity can impair ciliary function and mucocilliary clearance, thereby reducing overall drug retention. This effect has been observed with intranasal insulin [50]. Similarly, although higher viscosity increased residence time, metoclopramide absorption declined due to limited drug diffusion from the formulation [51]. An optimal viscosity of 100–400 mPa.S is considered acceptable.

The use of preservatives is an important formulation consideration, especially as regulatory agencies increasingly advocate for their removal. While they provide microbial control, longer shelf life, and preserved drug efficacy, preservatives can also cause nasal irritation, particularly with repeated dosing, allergic reactions, interactions with active compounds or excipients, and environmental concerns, which may outweigh their benefits [52,53]. Manufacturing and filling under clean conditions compliant with ICH guidelines can reduce or even eliminate the need for preservatives, especially when combined with preservative-free packaging systems and advanced dispensing technologies, such as preservative-free pumps. A decontamination step is often required, using heat, sterilizing gas, filtration, or radiation-based decontamination.

The nasal cavity also contains metabolizing enzymes such as carboxyl esterases, aldehyde dehydrogenases, epoxide hydrolases, glutathione S-transferases, and cytochrome P450 isoenzymes, which can reduce drug or peptide efficacy [54]. For instance, cytochrome P450 enzymes metabolize cocaine, nicotine, alcohols, and progesterone [41] while aminopeptidases and proteases degrade peptides such as calcitonin, insulin, and desmopressin. Although nasal first-pass metabolism is less extensive than hepatic or intestinal metabolism, it remains a relevant barrier [41]. Strategies to mitigate this include drug–drug combinations such as adding a vasoconstrictor to reduce systemic absorption into the bloodstream or an esterase inhibitor to improve prodrug stability [55]. However, this approach remains limited due to safety and regulatory challenges, and research in this area remains limited.

Variability in nasal physiology and anatomy, along with individual factors such as congestion, inflammation, and mucosal integrity, can significantly affect NtB drug delivery and must be considered. Nasal mucociliary clearance limits drug residence time, so drugs should ideally be available within 10 min of administration for efficient absorption, and nasal valve dimensions have been shown to influence intranasal oxytocin efficacy [56]. Standardized administration procedures and nasal examination can reduce variability due to anatomical differences [57]. However, the widespread distribution of trigeminal nerves in the nasal cavity provides a broader route than the olfactory pathway alone, helping to overcome these limitations.

Recent evidence extends beyond local nasal mechanisms, revealing physiological pathways that may influence peripheral signaling after intranasal delivery. The olfactory–trigeminal interface is a natural entry point for neurotropic viruses, mediated by receptor interactions in the nasal neuroepithelium, and underscores the accessibility of neuronal routes to the CNS. Importantly, emerging data demonstrate that CSF can communicate with peripheral neural structures via outflow along cranial and peripheral nerves. In a migraine model, CSF solutes reached the trigeminal ganglion and directly activated neurons [58]. More broadly, CSF flow along peripheral nerves has been reported [59], with mechanistic evidence supporting periaxonal CSF transport and functional signaling effects at and along nerves [60]. These pathways may guide the design and risk assessment of intranasal therapies, particularly regarding unintended peripheral signaling, and expand the physiological framework for interpreting intranasal strategies.

Thus, the NtB route presents a number of risks, which have been well described by Erdő et al. (2018) [8], including: variability in the concentrations achievable in different regions of the brain and spinal cord depending on the agent; a decrease in the efficiency of delivery of the drug with increasing molecular weight; potential partial degradation of certain therapeutic agents within the nasal mucosa; irritation of the mucosa; interference with drug delivery due to nasal congestion associated with colds or allergies; and the risk that the frequent use of this route may result in mucosal damage (e.g., infection, anosmia).

From a development perspective, these issues need to be anticipated and integrated into formulation design, dose selection, and the administration schedule.

Viewed as both a challenge and an opportunity, the NtB pathway holds significant promise for revolutionizing drug delivery and addressing unmet medical needs in neurology, psychiatry, oncology, and other therapeutic areas.

### 3.3. Evidence of Efficiency for the NtB Pathway

Researchers and drug developers have explored the NtB for decades. The earliest publication, from 1985, described transneuronal transport from the nasal epithelium to the rat olfactory system [61]. Later, Born et al. (2002) [62] showed that intranasal insulin, melanocortin, and vasopressin reached the CSF within 10 min in humans while plasma peaks occurred at 80 min. This breakthrough paved the way for multiple clinical studies using the NtB route.

NtB strategies have targeted various neurological disorders including Alzheimer’s disease, Parkinson’s disease, multiple sclerosis, epilepsy, and stroke [63]. There is no specific limitation on the pathologies that can be targeted using the NtB pathway, although our team and others have demonstrated that diseases affecting neurons are likely to be best suited to delivery through this route. In particular, it has been shown by our team that DCE given via the intranasal route in non-human primates was found mainly in neurons, and principally in cortical neurons [13].

Over 40 substances have been shown in animal studies to reach the CNS via the NtB pathway—often alongside the classical bloodstream-to-BBB route—using radiolabeling and microscopic techniques [61,64,65,66].

Preclinical and clinical trials have confirmed the safety, tolerability, pharmacokinetics, and preliminary efficacy of intranasal drugs in animals and humans, respectively, across conditions such as autism spectrum disorder, and schizophrenia to diabetes and migraine [31,43,65,67,68,69,70,71,72,73,74,75,76,77,78,79,80,81,82].

Table 1 summarizes the key findings from the intranasal delivery of small and large molecules in non-clinical and clinical studies.

Taken together, these data suggest that the NtB approach shows significant promise for drug delivery in neurological diseases, particularly those affecting neurons where conventional treatments often fall short. This pathway enables targeted delivery of neuroprotective agents, disease-modifying drugs, and gene therapies directly to neurons [83]. Studies with DCE [13,84,85] and a glucagon derivative [34,40] have demonstrated that drugs delivered via the NtB route achieve more efficient neuronal delivery than passive dispersion through brain tissue (e.g., extracellular matrix, lipids, etc.).

The pathway also supports the delivery of biologics, such as proteins, peptides, and nucleic acids, that cannot easily cross the BBB [86]. Advances in nanotechnology and drug delivery systems now allow their nasal delivery in animals, expanding therapeutic possibilities.

The NtB pathway is particularly promising when small doses suffice or if repeated administration achieves therapeutic at the site of action. High receptor potency or targeting neurons with repeated dosing can make NtB highly effective.

Although preclinical evidence supporting NtB delivery is extensive, clinical extrapolation has been restricted by formulation limitations, suboptimal device selection, or inadequate dose feasibility rather than any inherent failure of the pathway itself. These factors underline the importance of integrating industrial and CMC constraints early in development, a perspective that is central to the present review. Extrapolating findings from animals to humans will always be a crucial challenge.

It is important to note that, despite promising results, several preclinical intranasal programs have faced challenges in clinical extrapolation. For instance, multiple clinical trials of intranasal oxytocin for neuropsychiatric indications have yielded inconsistent clinical outcomes, despite robust preclinical evidence [57], with subsequent analyses pointing to heterogeneity in formulation, dose, and administration procedures. Similarly, intranasal insulin, initially showing encouraging cerebral distribution in human neuropsin gene [87] cognitive effects in early studies of Alzheimer’s disease, did not reproduce its efficacy in trials, largely due to differences in device type, emitted dose; and administration technique [88]. These examples underscore the need for integrated development approaches that fully account for pharmaceutical, device, and human-factor variables in NtB programs.

## 4. Strategy for Developing a Drug Using the Nose-to-Brain Pathway

### 4.1. Selection of the Chemical Substance and Its Suitability for the Nose-to-Brain Pathway: General Considerations and Application to Dodecyl Creatine Ester Nasal Spray

Developing a drug for the NtB pathway requires multiple considerations, ranging from chemical optimization to regulatory approval.

Candidate molecules should have properties that favor the NtB pathway over traditional routes, such as oral administration. Relatively hydrophilic (logP < 2) molecules face difficulties crossing membranes, particularly the BBB via the bloodstream, making them ideal for the NtB delivery. Additionally, the use of prodrugs that increase molecular weight as well as modifying lipophilicity can enhance solubility and membrane passage. A classic example is L-Dopa, a prodrug of dopamine, which cannot cross the BBB. For L-dopa, NtB delivery offers a significant advantage, bypassing the bloodstream and thereby avoiding hydrolytic degradation [89]. This strategy has been validated with other prodrugs, including γ-carboxylate-masked prodrug, γ-(4-acetoxybenzyl)-2-(Phosphonomethyl) pentanedioic acid [90], n-Butyl Nipecotate, the prodrug of nipecotic acid [55] and a phosphate ester prodrug of phenytoin [91].

Surprisingly, studies have shown no clear consensus regarding the relationship between physicochemical properties, such as molecular weight or LogD, and NtB efficacy [42,92]. This has led to the development of drug-vector conjugates to enhance NtB delivery. For example, conjugation with L-penetrating peptides substantially improved the brain distribution of molecules such as Exendin-4 and insulin [38,93,94,95,96]. Solubility in the nasal mucosa is also a limiting factor, due to the limited absorption capacity [8]. Also, compounds that are subject to cytochrome P450 metabolism and/or efflux transporters (P-gp or BCRP) may show reduced brain distribution when administered via the systemic route, making NtB delivery advantageous.

Drugs for NtB delivery must be stable in nasal mucus, non-toxic to the mucosa and olfactory or trigeminal nerves, and not adversely affect normal nasal function. These requirements ensure adequate brain penetration at therapeutic levels without local adverse effects.

### 4.2. Dodecyl Creatine Ester: A Creatine Prodrug for Neurological Diseases

#### 4.2.1. Creatine

Creatine is an organic compound derived from amino acids that plays a crucial role in cellular energy production. It is primarily synthesized in the brain, kidneys and liver from arginine, glycine, and methionine [97] and is also present in animal-derived foods such as meat and fish.

Creatine is converted intracellularly into creatine phosphate, a rapid energy reservoir that regenerates adenosine triphosphate (ATP), the cell’s primary energy source in mitochondria [98]. Phosphocreatine donates its phosphate group to adenosine diphosphate, forming ATP and creatine. This high-energy phosphate shuttle operates between mitochondrial production sites and cytosolic utilization sites [98].

Studies have suggested that creatine may protect cells by reducing oxidative stress and modulating signaling pathways. It shows neuroprotective potential, relevant for degenerative diseases, traumatic brain injuries, stroke, age-related loss of cognitive function, and more generally cognitive impairments [98,99].

Cellular uptake of creatine relies on a specific membrane transporter (SLC6A8/CT1/CRTR), which is highly expressed in skeletal muscle and kidney, and partially in the brain [97,100]. The transporter is saturated at physiological creatine levels [101], limiting efficient cellular uptake after oral supplementation [102,103] and thus innovative delivery methods of chemically optimized creatine are still needed.

#### 4.2.2. Creatine Transporter Deficiency

Creatine Transporter Deficiency syndrome (CrTD) is a rare X-linked genetic disorder caused by mutations in the SLC6A8 gene (Xq28) [104]. These mutations impair the creatine transporter (CrT), leading to deficient creatine uptake and disrupted energy metabolism. Clinically, CrTD presents severe global development delay, intellectual disability with marked speech impairment, autistic features, movement disorders, and seizures. Currently, no preventive or curative therapy exists for CrTD [105]. Patients do not respond to oral creatine monohydrate or administration of precursors such as glycine or arginine, treatments that are effective in guanidinoacetate methyltransferase (GAMT) and arginine–glycine amidinotransferase (AGAT) deficiencies, which affect creatine synthesis enzymes [104,106]. Hence, an effective therapy for CrTD remains an urgent unmet need.

#### 4.2.3. Amyotrophic Lateral Sclerosis

Amyotrophic Lateral Sclerosis (ALS), is a progressive neurodegenerative disease marked by the degeneration of upper and lower motor neurons in the brain and spinal cord, leading to muscle weakness, atrophy, paralysis, and respiratory failure [107]. Motor neurons are highly vulnerable to oxidative stress, due to their intense metabolic activity. Multiple mechanisms drive their degeneration, including mitochondria dysfunction (impairing energy production and increasing free radical), glutamate-dependent excitotoxicity, protein misfolding and aggregation (e.g., mutant SOD1), defective autophagy, and chronic microglia inflammation [108]. Energy imbalance and mitochondrial impairment have been well documented in ALS mouse models [109,110]. There is currently no preventive or curative treatment for ALS. Existing therapies, such as riluzole, which modulates glutamate release, and edavarone, an antioxidant and anti-inflammatory agent, provide only modest clinical benefits. These include extending life expectancy by a few months and improving patients’ quality of life. However, these treatments have limited efficacy and do not constitute a cure for ALS. Research is ongoing to develop more effective therapeutic strategies [111].

#### 4.2.4. Previous Therapeutic Attempts Using Oral Creatine Monohydrate in Creatine Transported Deficiency and Amyotrophic Lateral Sclerosis

Creatine exerts multiple biological effects relevant to neurodegenerative diseases, including neuroprotection, improved mitochondrial function, reduced inflammation, enhanced neuronal growth and survival, protection against excitotoxicity, and better muscle performance, and modified metabolism of creatine has been reported ALS. In SOD1 mutant mice, brainstem creatine levels decline from the presymptomatic stage (around 70 days). Creatine kinase and creatinine levels may serve as biomarkers for disease severity or progression, reflecting the underlying pathophysiological mechanisms of the disease.

Oral creatine monohydrate has shown beneficial effects in mouse models of ALS [112], but Phase 2 clinical trials failed to demonstrate significant efficacy [112,113]. Some studies in these patients, nonetheless, reported reduced N-acetylaspartate in the motor cortex [114], and a trend toward prolonged survival with supplementation [115].

Limited BBB permeability likely explains this discrepancy. The passive diffusion of creatine into the brain is restricted by a pre-existing concentration gradient, and CrT expression is heterogenous across neuronal populations. As a result, oral creatine supplementation is unlikely to markedly elevate neuronal creatine levels, regardless of CrT status, thereby limiting its therapeutic potential in ALS. Additionally, caffeine co-administration can reduce the effects of creatine and should be avoided [116] —this is less likely to be well controlled in Phase 3 trials. Finally, adherence to a placebo group is more difficult to control in Phase 3 trials of non-prescription compounds such as creatine monohydrate [117].

#### 4.2.5. Dodecyl Creatine Ester

Dodecyl creatine ester is a prodrug of creatine, chemically synthesized as an ester of creatine and dodecanol. Its LogP of 3.7 confers lipophilic properties, allowing it to cross biological membranes independently of the creatine transporter [12]. As creatine is hydrophilic (LogP −1.2) and dodecanol is lipophilic (LogP 5), DCE is amphiphilic.

A prodrug is designed to undergo biotransformation before exerting its pharmacological effect. When administered through routes that allow entry into the bloodstream, DCE is rapidly degraded into creatine and creatinine with an in vitro plasma degradation (t½) of 3 min [13]. As a result, any therapeutic central effect of DCE cannot be achieved following systemic exposure. This property renders DCE a particularly suitable candidate to demonstrate the relevance of the NtB pathway, as its primary pharmacological activity can only be achieved if the molecule reaches its neuronal target without first entering the bloodstream. Intranasal administration therefore provides a unique opportunity to preserve the integrity of the prodrug until it reaches the CNS. More generally, prodrug strategies combined with nasal delivery can enhance membrane penetration and improve stability or bioavailability [118]. Although the available evidence remains limited, several studies suggest that prodrugs may improve brain delivery via the NtB pathway while avoiding systemic exposure. For example, the prodrugs L-Tyrosine and nipecotic acid ester have shown better nasal absorption and brain distribution than their parent compounds [55,119]. Similarly, fosphenytoin increases solubility and nasal absorption of phenytoine [120].

Prodrugs can also bypass deficient transporters. In CTD, where the creatine transporter is inactive, a creatine prodrug like DCE—but not creatine itself—can reach neurons [12,84]. DCE delivers creatine to brain cells via ester hydrolysis, releasing active creatine and targeting neuronal energetic dysfunction.

**Figure 2:**
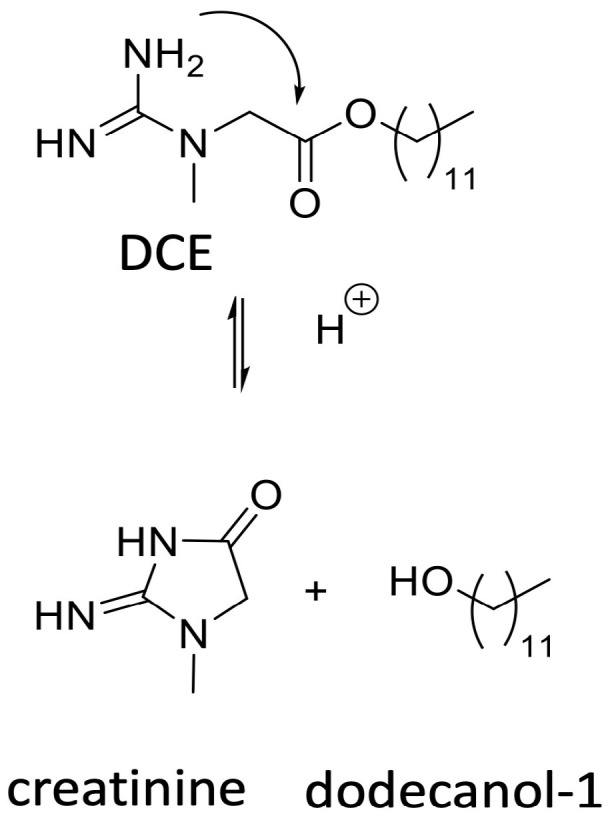
Chemical structure of dodecyl creatine ester (C_16_H_33_N_3_O_2_Cl) and of creatinine.

Although extrapolation from animal models to humans remains a critical step for any CNS-targeted therapy, the mechanisms observed with DCE in rodents [84] and non-human primates [13]—namely neural transport, cortical neuronal uptake and rapid prodrug activation—align with the biological principles already demonstrated in humans for other intranasally administrated compounds with central activity. Like all esters, DCE is inherently unstable in aqueous and biological environments. Besides hydrolysis, it can undergo intramolecular cyclization via the creatine guanidine group, forming creatinine (see Figure 2) that is also the cyclized terminal metabolite of creatine, influenced by both pH and temperature, occurring less at low pH and at 5–8 °C [121].

**Figure 3:**
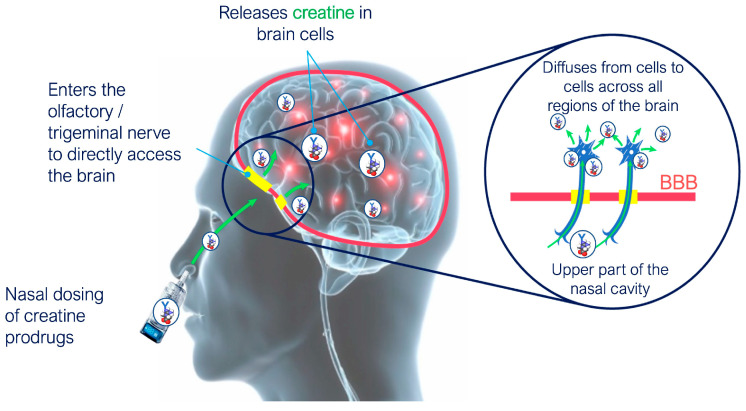
Mechanism of action of DCE as creatine prodrug.

A stabilizing formulation is therefore necessary to prevent DCE from chemical degradation. As a nasal spray, it is important to ensure appropriate dosing and comply with regulatory guidelines [122] on performance and microbiological quality [123]. Stability is achieved through an oil-in-water emulsion, where DCE remains suspended at the interface of both phases [13]. As a result, the DCE nasal spray has been shown to reach neurons effectively in non-human primates, delivering creatine and improving cerebral metabolism [13,85] (see Figure 3). It also produced beneficial behavioral effects in SLC6A6-KO mice, a model of CTD [84] and in 6-OHDA-intoxified rats [124]. The conserved organization of the olfactory and trigeminal pathways across species supports and although confirmation in humans will require dedicated evaluation, the biodistribution patterns in non-human primates (NHPs) are consistent with the well-established ability of intranasal administration to achieve central engagement in humans.

### 4.3. Development and Optimization of Specific Intranasal Formulations to Ensure Drug Stability, Facilitate Nose-to-Brain Delivery, and Improve Central Nervous System Bioavailability

Numerous publications and regulatory guidelines outline methods for developing nasal spray formulations. Key objectives include improving solubility and stability, maintaining a pH compatible with the mildly acidic nasal environment, and extending mucosal residence time through increased viscosity.

Formulation design begins with physicochemical characterization of the active compound, including its solubility, stability, and permeability, guiding the choice of excipients such as solubilizers and stabilizers. The formulation type is then selected accordingly: while aqueous solutions are often preferred for simplicity and control, suspensions or emulsions may be required for compounds with poor solubility. Emulsions can improve lipophilic drug solubility, protect from enzymatic or chemical degradation, enhance nasal absorption and brain delivery via the NtB pathway, and minimize local irritation compared with co-solvent systems.

To increase nasal residence time, viscosity enhancers (e.g., xanthan gum, carboxymethyl cellulose) or mucoadhesive polymers can be added. Prototypes are then optimized iteratively to balance stability, spray performance, and nasal tolerability. Both in vitro and in vivo evaluations are used in this stepwise approach to maximize drug stability and brain exposure via the NtB pathway while ensuring patient safety and regulatory compliance.

For solutions, emulsions, and suspensions, drug substance specifications should cover both the physical properties (e.g., shape, crystal structure, morphology) and particle size distribution (PSD), which are critical for reproducible formulations. PSD can be measured by laser diffraction for in-process control or using microscopy for specification monitoring. All methods should be validated for the requirements of Good Manufacturing Practice.

Emulsions are particularly favorable for NtB delivery as they can encapsulate both lipophilic and hydrophilic molecules [125]. The lipid phase facilitates penetration across nerve membranes, supporting efficient brain delivery, and can provide transient protection or controlled release. Excipients such as emollients and hydrating agents enhance local tolerance, preserving the integrity of the nasal mucosa. Kozlovskaya et al. (2014) demonstrated that particle-based formulations achieve higher direct brain transport (%DTP) than gels or solutions (60.7% vs. 59.5% vs. 36.6%) confirming improved efficiency for encapsulated compounds [42].

Nanoparticles offer several advantages, including their ability to carry molecules with diverse physicochemical properties and protect them from chemical and biological degradation in the nasal cavity. In addition, their compact morphology facilitates intracellular passage [93]. For example, wheat germ agglutinin (WGA) nanoparticles have been shown to enhance targeting of the olfactory region by the peptide NR2B9c, protecting neurons from ischemia-induced neurotoxicity due to abundant WGA receptors in the olfactory epithelium [115].

Compounds associated with nasal irritation, such as permeation enhancers, antioxidants, preservatives, flavoring agents, glycols or alcohols (e.g., transcutol) should generally be excluded from intranasal formulations [25]. If unavoidable, their concentration must be minimized. In contrast, humectants, such as glycerin (1–10%), sodium hyaluronate (<1%), sorbitol, or mannitol, can be added to protect the mucosa, typically in the aqueous phase of emulsions.

The pH of intranasal formulations should favor the non-ionized drug form for absorption while remaining close to the mildly acidic nasal environment to avoid irritation. Most nasal formulations are slightly acidic, typically pH 4–5, e.g., ketotifen fumarate sprays [126]. Some studies suggest that an optimal range of pH 4.5–6.5 is often recommended [54] and several studies suggest that a broader pH range (pH 3–10) can be used without cellular damage. Indeed, pH lower than 4 have been shown to be safe and tolerated [127], as demonstrated by FDA-approved sprays such as desmopressin, calcitonin, PATANASE^®^ (down around pH 3.5 for both), RYALTRIS^®^ (pH 3.7), and GENCYDO^®^ (pH 3.5–3.7). An acidic pH to enhance stability and maintain mucosal tolerability is therefore justified.

A key challenge in formulation development is transitioning from an experimental formulation to scalable, patient-ready formulations, as methods like ultrasonication are difficult to industrialize. Emulsion-based systems offer clear advantages: they are easier to administer, well tolerated, and more compatible with large-scale manufacturing processes than complex systems such as solid dispersions, liposomes, or particulate carriers [44].

For DCE, a chemically unstable drug for long-term nasal use, in our view five formulation characteristics are critical. First, stability of the active ingredient is the main priority, followed by local tolerance, formulation stability, sprayability, and loading, as devices can be adapted if needed. Stabilizing DCE, particularly as a creatine prodrug, is challenging: attempts with emulsions or solutions have failed to maintain stability, even at 5 °C [12,84]. The successful approach was an oil-in-water emulsion in which DCE remains under particles [13]. Its amphiphilic nature positions it at the oil-water interface, allowing surfactant concentrations below 1%. No preservative was used, and humectants and moisturizers enhanced nasal tolerability. This approach achieved long-term stability at 5 °C and excellent tolerability in toxicity studies [13].

### 4.4. Manufacturing Process Development and Quality Validation of the Intranasal Spray Formulation

Nasal spray manufacturing follows key regulatory requirements [122,123]. The main considerations are summarized below:

- Sterility is not mandatory if microbiological quality complies with Pharmacopeial standards [128] as the nasal cavity hosts diverse microflora. Production must nonetheless occur in a suitably controlled cleanroom environment.
- The choice between single-dose and multi-dose delivery systems is essential. Single-dose devices deliver a precise amount, which is ideal for short treatments or controlled drugs and minimizing contamination risks. Multi-dose formats are preferred for long-term treatments or frequent dosing.
- The delivery volume is limited by nasal cavity size—typically <200 μL/nostril in adults and <100 µL/nostril in children [129]—to avoid drooling and nose fullness sensation, mucosal irritation, bacterial growth, and disruption of ciliary function.
- Ensuring consistent, reproducible dosing is a major challenge, particularly in self-administration. Device design, nasal air flow, and compliance all affect deposition and efficacy. Education, training, and intuitive devices are critical to ensure correct use and adherence, and reproducibility can be evaluated through pharmacokinetic and/or pharmacodynamic studies. Variability of drug brain exposure after nasal dosing is generally comparable to other routes, although it can be slightly higher in some cases. For example, midazolam shows a brain exposure variability of 15% (nasal administration) versus 24% (oral administration) [130]. For sumatriptan these are equivalent for both routes [131]. Ketamine shows variability ranging from 47 to 76% after nasal administration, versus 31 to 35% after oral administration [132].
- In line with nasal spray guidelines [122,123] key tests include: drug identification, assay, impurities, delivery performance (mean and uniformity per actuation), spray pattern and plume geometry, microbial limits, preservative content, dose count, droplet (to monitor particles <10 µm potentially reaching the lungs) and particle size distribution for suspensions).

### 4.5. Nasal Administration Devices and Techniques

#### 4.5.1. General Considerations

Selecting the appropriate type of device for intranasal administration is a critical step in developing a nasal product. It must maintain drug stability and ensure accurate, reproducible dosing in line with EMA and FDA requirements [122,123].

Dosing techniques depend on the device and should enable effective delivery of the drug to the intended nasal region. Numerous devices are available, each with specific technical and functional features influencing their suitability for NtB delivery. Key factors include the efficiency of targeting a particular region (e.g., olfactory vs. respiratory), compatibility with the formulation, deposition performance, patient usability, and regulatory compliance.

Physicochemical and aerodynamic properties, such as spray pattern, plume geometry, droplet size, and actuation force, determine regional deposition and brain biodistribution. A narrow plume promotes olfactory deposition, whereas broader angles favor trigeminal uptake. For example, Impel NeuroPharma’s POD^®^ system achieved deep olfactory delivery via gas-propelled plumes [133] while Kurve Technology’s Vianase adjusts electronic waveforms in real-time to optimize posterior nasal targeting [134].

In vitro studies using nasal replicas have confirmed that the cone angle of the spray has the strongest effect on posterior nasal deposition [135], followed by plume ovality, droplet size, velocity, and size distribution uniformity. Droplet size, expressed as median diameter of the droplet volume, is key to retention: 10–50 µm favors nasal deposition whereas >50 µm may be cleared rapidly by mucociliary action and <10 µm risks pulmonary exposure. Thus, formulations must be matched with device performance to ensure optimal residence time and efficient transport via olfactory and/or trigeminal pathways.

Representative examples of nasal delivery devices are described in Table 2.

Nasal delivery devices must comply with regional regulations on safety, efficacy, quality, and usability, including human factors (per FDA and EMA guidelines). This is particularly important for preservative-free systems, which must maintain microbiological integrity throughout the product’s lifecycle. For chronic or home use, ease of operation is critical: device should deliver accurate, repeatable doses, minimize handling errors, and provide clear instructions though appropriate actuation force, device priming, dose counters, and feedback mechanisms (e.g., audible or visual cues). In this way patient adherence will be improved and misuse will be reduced. Devices must also accommodate patient variability, including age- or disease-related limitations in dexterity, making user-centered design essential for both regulatory approval and therapeutic success.

When combined with a medicinal product, the device may form an integral drug-device combination (iDDC) [136] regulated under the medicinal product framework (Directive 2001/83/EC or Regulation (EC) No 726/2004 of the European Parliament) [137]. In this case, the device must meet relevant General Safety and Performance Requirements (GSPRs) of Annex I of the Medical Device Regulation (MDR 2017/745). Similarly, in the United States, such a product would be considered a single-entity combination product under FDA’s combination product regulations (21 CFR Part 3). If the drug provides the primary mode of action, CDER has primary jurisdiction, while the device constituent part must still meet applicable medical device requirements, including those of 21 CFR Parts 820, 801 and 803. Thus, in both jurisdictions (i.e., Europe and the United States), although the overall product is regulated as a medicinal product, the device component must meet the essential/device-specific requirements of the respective regulatory framework.

If supplied separately or co-packaged, the device falls under the medical device framework and must be CE marked. For nasal sprays, which are non-sterile, the device is typically class I non-sterile, which requires declaration of conformity in the marketing authorization dossier.

FDA [128] and EMA [122] guidelines define the tests that are required for clinical development of an iDDC. For non-pressurized multi-dose sprays, key evaluations include physical characterization, minimum fill justification, extractables/leachables, and particle and droplet size distribution. Microbiological integrity and device robustness must also be demonstrated for preservative-free systems throughout their lifecycle. Usability studies are essential—particularly for chronic or home use—to confirm dosing accuracy, repeatability, minimal handling errors, and clarity of instruction. Further assessments should verify dose uniformity throughout the shelf life of the product, actuator or mouthpiece deposition, effect of shaking, priming and re-priming, cleanliness, and performance under temperature cycling.

#### 4.5.2. Nasal Administration Device for Dodecyl Creatine Ester Formulation

Various preservative-free nasal spray devices were evaluated for the development of the DCE formulation. The main objectives were to assess plume geometry, spray ovality, delivered dose (accuracy and precision), useability, and preservative properties.

Performance varied between pumps due to the specific characteristics of the formulation. The first selection was based on plume geometry and ovality—devices with a plume angle > 10° and good ovality (close to 1, indicating homogeneous spray distribution) were chosen, assuming they could reach the olfactory and trigeminal nerves regions. A second selection then focused on spray reproducibility and uniformity of the delivered dose, both within a single container and between containers, in line with European and US guideline for nasal spray [122,123]. Additional tests on actuation force, usability and microbiological control allowed the final pump to be selected. For the pharmaceutical development of DCE, the VP7/CPS pump from Aptar was selected for its preservative-free, multi-dose capabilities and for its ease of use and reproducible delivery performance, especially with a relatively viscous formulation (350 mPa.s).

### 4.6. Pharmacokinetic Studies: Assessing the Extent of the Nose-to-Brain Pathway—General Considerations, Case Examples, and Application to Dodecyl Creatine Ester

The NtB pathway refers to the direct transport of a drug from the nasal cavity to the brain, bypassing the systemic circulation, rather than entering the bloodstream first and crossing the BBB. Distinguishing between these pathways is essential to assess the contribution of the NtB delivery route versus systemic exposure following conventional oral or intravenous administration. Non-clinical pharmacokinetic studies have aimed to show that nasal administration enhances brain distribution compared to systemic routes. Protocols have compared brain uptake and bioavailability between administration routes to determine the efficiency of the NtB route. Key parameters include Drug Targeting Efficiency (%DTE) and direct NtB percentage (%DTP) [42].

#### 4.6.1. %DTE and %DTP

%DTE measures brain exposure after intranasal versus systemic administration. Values > 100% indicate more efficient brain delivery via the intranasal than the systemic route. It is calculated as follows:

%DTE = (AUCbrain/AUCblood in)/(AUCbrain/AUCblood iv) × 100

where AUC is the area under the curve of the drug concentration versus time, for brain and blood samples.

Many compounds have shown %DTE >100%, demonstrating effective NtB delivery (e.g., morphine in rats [67]), and the type of formulation has been shown to influence %DTE: gel > particle > solution (518%, 475%, and 370%) [42].

%DTP (direct NtB transport percentage) estimates the percentage of the dose reaching the brain via the NtB pathway relative to total brain exposure, and is calculated as follows:

%DTP = (Bin − Bx)/Bin × 100

where Bin is the brain AUC after intranasal dosing, BX is the fraction delivered via systemic circulation calculated as: Bx = Biv × Pin/Piv (where Piv is blood AUC following intravenous administration, and Pin is the blood AUC following intranasal administration). As described for %DTE, the type of formulation also affects %DTP: particle < gel < solution (60.7%, 59.5%, and 36.6%).

These parameters have shown in animals that a substantial fraction of drugs reach the brain via the NtB, often >50% of total brain delivery [42] (e.g., for zonisamide %DTP is approximately 49% [69]). Extensive pharmacokinetic studies require large numbers of animals and multiple brain/plasma samples, making them complex, costly, and challenging in genetically modified models, raising 3Rs (replacement, reduction, refinement) concerns.

Beyond these practical considerations, %DTE and %DTP also rely on methodological assumptions that restrict their interpretation. These metrics require invasive sampling and therefore remain limited to preclinical models; they cannot be applied in humans and do not directly isolate the contribution of individual transport pathways. As a result, these parameters should be viewed as preclinical tools for comparing formulations or studying mechanisms rather than as predictors of human performance.

High %DTE and %DTP values have been shown for curcumin formulations: solution (433% DTE, 77% DTP) and docosahexaenoic acid (DHA) microemulsion (615% DTE, 97% DTP) [138]. Also, imaging techniques (gamma scintigraphy with 99mTc [139] or 125I [39]) help to track NtB delivery and calculate %DTE and %DTP.

#### 4.6.2. Enhanced Brain Penetration for Various Drugs Following Intranasal Administration

Several medicines have demonstrated enhanced brain delivery via intranasal administration through the NtB pathway. Midazolam is lipid-soluble and rapidly absorbed, with fast onset and better bioavailability when administered via the intranasal route compared to intravenously [140,141]. Talinolol demonstrated higher brain and CSF delivery in rats after intranasal perfusion than following IV infusion based on %DTE [70]. A chitosan-based buspirone intranasal formulation achieved 2.5-fold greater brain uptake than either IV or a standard intranasal formulation [142]. Intranasal deferoxamine increased frontal cortex targeting 271-fold compared to IV dosing, suggesting potential for ischemic stroke therapy [143]. Didanosine delivered via the intranasal route enhanced CSF and brain exposure relative to IV, indicating direct NtB transport [144], while stavudine showed comparable brain concentrations after intranasal and IV administrations [145]. Progesterone and pregnenolone exhibited higher uptake in the olfactory bulb, hippocampus, and hypothalamus after intranasal administration compared to IV [146]. Enhanced cerebral distribution following intranasal administration has also been reported for duloxetine, erythropoietin, estradiol, interferon beta, hexarelin, risperidone, rivastigmine, ropinirole, sumatriptan, and venlafaxine [142]. Bromocriptine formulations were more effective via nasal than IV, consistent with direct NtB transport, as demonstrated by gamma scintigraphy imaging in mice [147].

#### 4.6.3. Cerebrospinal Fluid

The relevance of measuring drug levels in CSF as a surrogate for brain concentrations is limited. A drug can reach the CSF via CNS exposure through the NtB route, with peak levels as early as 15 min post-nasal administration [7], but CSF can clear compounds from the brain, including anions, xenobiotics, amino acids, and prostaglandins, particularly at the blood-meningeal barrier. Consequently, CSF concentrations do not reliably reflect brain tissue levels [148], as observed for antiretrovirals such as tenofovir, emtricitabine, raltegravir, maraviroc, and zidovudine [149].

#### 4.6.4. Other Pharmacokinetic Considerations

Determining the total amount of a molecule reaching the brain after intranasal administration remains challenging. This limitation also applies to other administration routes because of continuous drug influx and clearance from the brain. Thus, measured concentrations reflect only transient levels, not the total dose delivered. Similarly, a concentration-time AUC does not provide the actual dose delivered to the brain. Although this may be frustrating for drug developers, it mirrors the same challenges encountered in the development of CNS-targeted drugs delivered via conventional routes, such as oral or intravenous administration. Pharmacokinetic studies therefore compare brain and blood concentrations after intranasal versus other routes, providing a relative measure of brain delivery rather than an absolute quantification.

Intranasally administered drugs also undergo less metabolism in the nasal cavity and brain than would occur in the blood and liver. For example, a GLP-2 derivative has been shown to be transferred via the olfactory bulb and the principal sensory trigeminal nucleus without metabolism [34] and labelled leptin shows lower degradation in rat brain than plasma, supporting direct leptin NtB transport [150].

#### 4.6.5. Preclinical Studies

Preclinical studies in a range of animal models have allowed complementary evaluation of cerebral pharmacokinetics and therapeutic efficacy in neurological disorders. Rodents provide a rapid first step to characterize pharmacokinetic parameters for IV, oral, and nasal routes of administration [151] and to assess pharmacological effects in models of neurodegenerative or neurological diseases [67,152]. However, the small size of rodent brains limits detailed regional or cellular analyses, especially at low concentrations or after a single dose. Larger animals, particularly NHPs, enable more translational and relevant data, allowing targeted sampling of brain structures, including neuronal, glial, and endothelial compartments, for cellular-level analysis [153]. Non-invasive imaging such as positron emission tomography (PET) or magnetic resonance imaging (MRI) can provide complementary dynamic in vivo data on cerebral activity and biodistribution. Optimizing therapeutic efficacy requires the refining of administration parameters, such as dose and frequency, using data generated throughout preclinical development.

Regulatory studies require plasma data obtained according to Good Laboratory Practice, but from a scientific standpoint such data may be of limited scientific relevance, particularly for prodrugs that are inherently unstable in plasma [7].

#### 4.6.6. Considerations on Clinical Studies

Complementary approaches, such as PET/single photon emission computed tomography (SPECT) imaging or CSF sampling, can offer additional insights into CNS exposure in NHPs [87] and humans. However, these methods cannot isolate the specific contribution of the NtB pathway.

Directly measuring drug concentrations within the human brain remains highly challenging. Imaging techniques typically require radiolabeling the molecule, often using short-lived isotopes such as ^11^C or ^18^F. This approach is only feasible for a limited range of compounds and requires advanced radiochemistry infrastructure, including on-site cyclotron facilities. While CSF sampling is less invasive, it does not reliably reflect brain parenchymal concentrations [148], as CSF composition changes rapidly and represents a dynamic equilibrium rather than true tissue exposure.

These limitations are not unique to the NtB pathway–they apply to all CNS drug delivery strategies. However, developers focusing on NtB modalities may encounter these challenges more frequently, perhaps because this pathway represents a less conventional approach. Collectively, these tools provide a translational framework for assessing CNS exposure, albeit with inherent methodological constraints.

#### 4.6.7. Example of Dodecyl Creatine Ester

Several pharmacokinetic studies of DCE have been conducted using SLC6A8 KO mice and wild-type cynomolgus monkeys after nasal and/or IV administration.

Creatine prodrugs pose two main challenges: first, they are generally unstable in biological media, and second, creatine, the active metabolite, is already abundant in blood and brain, complicating quantification [154]. Accurate monitoring requires enrichment of stable isotopes (e.g., ^2^H, ^13^C or ^15^N) and LC-MS/MS for quantification [13,84] or radiolabeling (e.g., ^14^C or ^3^H).

After intranasal administration of ^2^H and ^13^C-labelled DCE, blood levels have been shown to be undetectable in mice and <1 ng/mL in NHPs, reflecting rapid metabolism (degradation t½ of 3 min in plasma and around 15 min in nasal mucus) [13]. In contrast, repeated nasal dosing NHPs led to neuronal uptake of labelled-DCE and labelled-creatine, mainly in the cortex, striatum, hippocampus, and white matter [13]. Intravenous administration yielded undetectable plasma levels in mice and levels that were barely detectable in NHPs, with labelled creatinine predominating, preventing pharmacokinetic parameter estimation for DCE [13]. Consequently, DTE% and DTP% could not be calculated.

These results help identify the precise site of action in the brain, a factor that is critical yet often understudied and frequently overlooked when selecting the most appropriate disease target. Our work, along with that of others [34,40], suggest that the term “Nose-to-Brain” is overly broad, and that the term “Nose-to-Neurons” (NtN) is more accurate. This is based on the drug travelling along the olfactory and trigeminal nerves and, through synaptic transmission, preferentially targeting neurons over other brain structures [30]. These findings have translational relevance due to the conserved organization of the olfactory and trigeminal pathways across species, supporting their applicability to human intranasal delivery. Moreover, human confirmation requires clinical evaluation, but the biodistribution and metabolic profiles observed in NHPs align with the well-established ability of intranasal administration to achieve central engagement for other compounds.

### 4.7. Toxicological Issues to Consider

#### 4.7.1. General Considerations

Safety studies are required before human trials to assess (i) systemic toxicity, since a part of the drug may enter the bloodstream, and (ii) local tolerance, especially for the nasal route.

Rats are conventionally used for these studies, with doses adjusted by volume (typically 10–40 µL daily, possibly divided into multiple administrations). For non-rodents, Beagles or NHPs are preferred, usually Beagles due to their availability and easier handling. Their nasal cavity volume (around 20–30 mL [155]) facilitates dose extrapolation to the human situation and the same device as the one intended for humans can often be employed. Dose adjustments are achieved by varying drug concentrations or the number of actuations (when the delivered volume is fixed).

Beyond standard clinical observations, biological assessments, macroscopic exams, and targeted histopathology should include the spleen, stomach, kidneys, liver, and tissues associated with nasal administration and the NtB pathway. Microscopy should also cover the nasal cavity, salivary glandes, pharynx, larynx, tongue, trachea, olfactory bulb, and other cerebral structures.

Toxicological studies should use the formulation and delivery system intended for humans, with excipients at concentrations identical or very close to the final product.

For chronically administered intranasal products, long-term safety evaluation typically includes assessment of local tolerability, epithelial integrity, as well as monitoring for potential immune or inflammatory responses, particularly after multiple dosing. These requirements are standard for all nasal formulations and are applied case-by-case depending on the duration of treatment. In our view, these aspects should be integrated into early formulation and CMC considerations to ensure product-specific safety. Toxicological studies in rodents and larger animals should incorporate these measurements with neurological assessments as components of the safety pharmacology package.

#### 4.7.2. Extrapolation Dosage from Animal to Human for First-in-Man Studies

In animals, drugs reach the brain via the olfactory and trigeminal pathways, similar to intranasal delivery in humans. However, the olfactory surface area is relatively larger in animals: around 50% of the nasal cavity in rodents [156] versus 5% in humans. The olfactory–respiratory epithelium ratio is approximately 50:50 in rodents, compared to 2:98 in humans, raising translational concern. NHPs provide a closer approximation (around 10% olfactory area [156]) though the human olfactory bulb represents only 0.064% of brain mass, far smaller than in rodents (4–20% [7]). In rats, Labrador retrievers, and humans, nasal epithelium surface areas are, respectively, 14 cm^2^, 210 cm^2^, and 160 cm^2^ [8]. Species also differ in CSF turnover, olfactory bulb protein binding, and trigeminal nerves distribution [7]. These anatomical and physiological differences limit the translatability of rodent data. For humans, drug developers should target both olfactory and respiratory epithelia to engage the NtB pathway and consider both the dosing regimen and target cell types, noting that neurons are more developed in humans.

For nasal dosing, translation therefore relies on the use of relevant species, often non-rodents, to determine the No Observed Adverse Effect Level (NOAEL) [122] which inform human exposure. Translation may involve allometric scaling or pharmacokinetic/pharmacodynamic (PK/PD) and physiologically based pharmacokinetic (PBPK) modelling, though NtB-specific models remain limited. Blood concentrations are often used but may poorly reflect brain exposure for molecules acting via the NtB pathway. FDA Guidance [157] provides methods for converting animal NOAELs to human doses (mg/kg, mg/m^2^, or mg/area of application). For nasal drugs with possible local toxicity, dose per area of application (e.g., area of the nasal cavity) complements weight-based calculations, improving systemic and brain toxicity assessment. Phase 1, first-in-man clinical studies are critical to detect nasal-specific adverse effects such as irritancy, unpleasant taste, smell change, headache, dizziness, or nausea, which cannot easily be assessed in non-human species. Conducting early trials in vulnerable patients may also prevent the collection of such data potentially causing issues in later clinical trials.

#### 4.7.3. Therapeutic Effects Measurable in Preclinical Studies and Their Clinical Translatability

Pharmacodynamic studies have confirmed central target engagement and quantified molecular and cellular effects. In animals, neurotransmitter levels (e.g., dopamine, serotonin) can be measured by microdialysis [158] or post-mortem using LC-MS/MS to detect the drug, metabolites, or neurochemical changes. Gene and protein expression analysis (qPCR, Western blot, or immunohistochemistry) have further characterized molecular effects. PET receptor occupancy studies enable in vivo quantification of target binding [159] and downstream markers such as pERK, pCREB, cAMP, or IP3 indicate receptor activation and signal transduction. When validated, CSF or plasma biomarkers provide minimally invasive surrogates for CNS activity [160].

Functional assessments address physiological and behavioral outcomes. In preclinical models, validated paradigms of motor, cognitive, or sensory function have been used to evaluate efficacy, while electroencephalogram (EEG) or local field potential (LFP) recording have been used to assess neural circuit modulation [161]. In clinical studies, functional MRI (fMRI) [162], PET, quantitative EEG (qEEG) [163], and standardized scales, such as the ALS Functional Rating Scale (revised) (ALSFRS-R) [164] an serve as primary or secondary endpoints.

### 4.8. Regulatory Aspects

Regulatory approval of CNS-acting intranasal products is similar to that for other nasal formulations, as no dedicated regulatory framework currently exists for therapies specifically targeting the NtB pathway. Notably, regulatory agencies such as EMA and FDA do not require direct quantification of brain delivery in humans. Instead, approval is based on the assessment not only of systemic pharmacokinetics, demonstrated clinical efficacy, pharmacodynamic evidence, and safety, but also quality according to the nasal spray regulatory requirements of the EMA [122] and FDA [123] and the performance and safety of the device–formulation combination (as outlined in General safety and performance requirements [GSPRs], Annex I of the Medical Device Regulation (MDR 2017/745)).

Bioequivalence assessments for generic intranasal products follow established guidelines on bioequivalence (based on the systemic pharmacokinetics, although of poor relevance in this case) and are not unique to NtB approaches. As a result, the feasibility of an NtB-based development program depends on the same regulatory principles that govern conventional nasal products.

From a commercial perspective, market adoption is facilitated by the convenience of this route of administration, including in elderly patients and children, and ultimately relies on demonstrating meaningful therapeutic benefit rather than on quantifying drug concentrations within the brain or meeting other requirements associated with a hypothetical NtB-specific regulatory category.

## 5. Conclusions

The development of drugs designed to exploit the NtB pathway, exemplified by DCE designed to address CrTD and mitochondriopathies, represents a biological coherent and translationally motivated approach that warrants more clinical investigation. This review highlights both the opportunities and challenges associated with this direct brain delivery approach.

Unlike oral or IV routes, the NtB approach uses the olfactory and trigeminal nerves to deliver drugs to the CNS while minimizing systemic exposure. It predominantly targets neurons, offering a clear advantage for neuron-centric disorders.

The NtB pathway enables the delivery to the CNS of molecules that are not able to cross the BBB and/or are unstable in the bloodstream, achieving higher brain concentrations and fewer peripheral effects. It is suitable for a wide range of small molecules, peptides, and even for proteins, opening opportunities for neurologic and psychiatric diseases. Its non-invasive nature also improves patient comfort and thus compliance.

The main limitation of the NtB pathway is the restricted drug quantity reaching the brain, making this route best suited for potent drugs, for supplementation of endogenous compounds, and for neuron-centric diseases. Data from our team on DCE [13,84] suggest that treatment duration may be more critical than dose level for efficacy. Physicochemical properties, formulation, and anatomical variability all affect the efficiency of delivery through the NtB pathway, while local safety must also be carefully monitored during chronic treatment.

Nasal variability and mucosal integrity may influence absorption, but these factors are only slightly greater than for other routes, e.g., oral administration, and can be mitigated through the use of well-defined procedures and patient training. Another issue for NtB administration is local irritation or toxicity. It is therefore crucial to optimize excipient selection and formulation, particularly for chronic treatments such as DCE for CTD or other neurologic mitochondriopathies.

For DCE, the NtB route uniquely enables creatine delivery to neurons, bypassing the systemic circulation [13]. Preclinical results in CrTD mice and wild-type monkeys demonstrate therapeutic benefit [84,85] and neural uptake [13], providing a rationale for possible extrapolation to humans. Using optimized nasal devices and administration protocols would enhance compliance and outcomes.

As with any intranasal modality intended for repeated administration, long-term safety must include the evaluation of both local tolerance and systemic exposure [10].

Overall, the NtB route should be viewed neither with excessive optimism nor undue skepticism, but as a scientifically grounded modality whose success depends on the rigorous integration of pharmacology, formulation science, and industrial constraints.

## 6. Future Directions

Few approved drugs rely solely on the NtB route, insulin being one example. Others, such as oxytocin (Prader-Willi) and DCE (CrTD and related diseases) are under development. Many compounds (e.g., morphine, sumatriptan, esketamine) reach the brain via mixed mechanisms combining systemic and direct transport. The limited number of NtB drugs also reflects CMC complexity.

Given the convenience of nasal administration, it is reasonable to ask why more drugs are not available to exploit the NtB pathway. Key limiting factors include the compound’s physicochemical properties and the dose needed for efficacy. Moreover, drug product development, notably CMC, for the NtB pathway is more complex than for conventional oral tablets or capsules, demanding stricter oversight.

The exact site of action in the brain is critical, and the term “Nose-to-Neurons” (NtN) better reflects the concept [34,40]. This route should be viewed not merely as a way to deliver drugs to the brain, but as a strategy to target neurons directly, highlighting its relevance for neuronal rather than non-neuronal disorders.

Taking these considerations into account, drug development exploiting this pathway holds strong promise as the field matures. Unlocking its potential will depend on rational drug design, optimized nasal formulations, and targeting neurons via both the olfactory and trigeminal nerves, while ensuring local safety, scalability, and industrial feasibility, which are factors that are critical for successful development.

## Figures and Tables

**Table 1:** Intranasal delivery of small and large molecules: key findings.

Compounds	Function	Species	Key Findings
Small molecular weight (<10 kDa)			
Morphine	Centrally acting analgesic	Rat	Morphine follows the NtB pathway, confirmed by plasma-to-brain AUC ratio Brain levels at 5 and 15 min post-intranasal dosing match intravenous administration Despite lower plasma levels, intranasal delivery shows an early brain distribution advantage [67]
Cocaine	Centrally acting stimulant	Rat	Cocaine produces effects within minutes of nasal use, before bloodstream detection, indicating an alternative brain entry pathway [68]
Zonisamide	Antiepileptic drug	Mouse	IV route: elacridar did not affect plasma zonisamide but tripled brain exposure IN route: no significant plasma or brain changes, suggesting limited BCRP influence Findings indicate zonisamide uses the NtB pathway for brain delivery [69]
Talinolol	Cardioselective β1-blocker	Rat	IN dosing results in greater delivery to the brain and CSF compared to IV [70]
Ketamine	General anesthetic and NMDA receptor antagonist	Human	Blood concentrations of ketamine were lower after IN administration than IV, despite achieving a comparable pharmacological effect, suggesting reduced peripheral exposure with similar CNS response IN dosing resulted in a lower proportion of responders compared with IV, highlighting the variability in response among individuals [43]
Prodrug of levodopa (L-dopa)	Precursor to dopamine, adrenaline, noradrenaline	Rat	IN administration of L-dopa results in higher concentrations in CSF and olfactory bulb compared to IV delivery This suggests that the butyl ester can reach the CSF or olfactory bulb through the NtB pathway [71]
Domperidone	Dopamine antagonist medication	Cynomolgus monkey	PET imaging of [18F] fallypride detected the compound in the caudate and putamen This indicates its transport to the brain through the NtB pathway [31]
Oxytocin	Peptide hormone and neuropeptide	Human	Limited ability to cross the BBB IN administration enables oxytocin to reach the brain via the NtB pathway and has been shown to improve trust, empathy, and emotional recognition, including in individuals with autism spectrum disorders [72,73,74,75]
Leucine- enkephalin	Peptide neurotransmitter	Rat	Reach the brain only when formulated as nanoparticles and administrated intranasally Showed no detectable peripheral exposure or activity [76]
Insulin	Peptide hormone	Human Rat Mouse Non-human primate	Dysregulation is linked to metabolic disorders (obesity, type 2 diabetes) and neurodegenerative diseases (AD) IN insulin administration showed minimal systemic absorption and predominantly reaches the brain via the NtB pathway [77,78,79]
High molecular weight (>15 kDa)			
Leptin	Protein hormone	Rat	IN leptin delivers directly to the CNS Significantly reduces food intake [80]
Nerve growth factor (NGF)	Neurotrophic factor Neuropeptide	Rat Mouse	IN administration delivers to the brain reaching the olfactory bulbs Showed neuroprotective effects [65,81]
Plasmid DNA	Extrachromosomal DNA	Rat (cell culture)	IN administration successfully delivers intact cells to the brain Mesenchymal stem cells and glioma cells reach the brain within 1 h Suggests IN delivery could enable stem cell therapies for CNS disorders [82]

AUC, area under the curve; BCRP, breast cancer resistance protein; CNS, central nervous system; CSF, cerebrospinal fluid; IN, intranasal; IV, intravenous; NtB, Nose-to-Brain; PET, positron emission tomography.

**Table 2:** Summary of nasal device characteristics and target brain areas.

Device	Format	Formulation Type	Target Region(s)	Mechanism or Feature	References (URL Accessed on 19 December 2025)
Aero Pump	Multidose	Aqueous	General nasal cavity	Simple preservative-free spray	https://www.aeropump.de/en/products/nasal
Aptar VP7 or CPS	Uni/ Multidose	Aqueous	Olfactory & trigeminal	Preservative-free, consistent metering	https://aptar.com/en-us/products/pharmaceutical-cps-technology-platform
Gerresheimer	Multidose	Aqueous	General nasal cavity	Low actuation force	https://www.gerresheimer.com/fileadmin/user_upload/user_upload/primary-packaging/download/bottles-containers/Gerresheimer_Snap-on.pdf
Impel POD^®^	Unidose	Liquid	Olfactory bulb	Gas-propelled delivery, deep targeting	[133]
Kurve Vanase	Electronic	Liquid/ Powder	Upper posterior	Controlled waveform spray	[134]
Nemera	Multi/ Unidose	Aqueous	ENT/Olfactory	Range of nasal delivery options	https://www.nemera.net/products/ear-nose-throat/multidose-pumps/sp270-sp370/
Optinose OptiMist™	Breath-powered	Aqueous	Upper posterior	Patient-actuated via exhalation	https://optinose.com/exhalation-delivery-systems/technical-overview
Unither	Unidose	Aqueous	Variable	Simple, scalable, preservative-free	https://www.unither-pharma.fr/wp-content/uploads/2021/11/PFMD_Unither_1-page_EN.pdf

## Data Availability

Sources for the data reported in this article are available publicly or will be made available to researchers who provide a methodologically sound proposal by application to the Corresponding Author.

## Abbreviations

The following abbreviations are used in this manuscript:

AGAT	Arginine–glycine amidinotransferase
ALS	Amyotrophic lateral sclerosis
ALSFRS-R	Amyotrophic lateral sclerosis functional rating scale (revised)
AUC	Area under the curve
ATP	Adenosine triphosphate
BBB	Blood–brain barrier
BCRP	Breast cancer resistance protein
CMC	Chemistry, Manufacturing, and Controls
CNS	Central nervous system
CrT	Creatine transporter
CrTD	Creatine transporter deficiency
CSF	Cerebrospinal fluid
DCE	Dodecyl creatine ester
DHA	Docosahexaenoic acid
DTE	Drug targeting efficiency
DTP	Direct NtB percentage
EEG	Electroencephalogram
fMRI	Functional MRI
GAMT	Guanidinoacetate methyltransferase
GLUT	Glucose transporter
GRAS	Generally recognized as safe
GSPR	General safety and performance requirement
iDDC	Integral drug-device combination
IN	Intranasal
IV	Intravenous
LFP	Local field potential
MCT	Monocarboxylate transporter
MDR	Medical device regulation
MRI	Magnetic resonance imaging
MRP	Multidrug resistance-associated protein
NHP	Non-human primate
NOAEL	No observed adverse effect level
NORD	National Organization for Rare Disorders
NtB	Nose-to-brain
NtN	Nose-to-neurons
PBPK	Physiologically based pharmacokinetic modelling
P-gp	P-glycoprotein
PEG	Polyethylene glycol
PSD	Particle size distribution
PET	Positron emission tomography
qEEG	Quantitative EEG
SPECT	Single photon emission computed tomography
WGA	Wheat germ agglutinin
